# Ultrasound and Enzyme-Assisted Development of Advanced Ingredients from Rowanberry (*Sorbus aucuparia* L.) Pomace and Its Application in Bread

**DOI:** 10.3390/foods15091494

**Published:** 2026-04-24

**Authors:** Simona Ražanaitė, Laura Jūrienė, Rita Kazernavičiūtė, Michail Syrpas, Petras Rimantas Venskutonis

**Affiliations:** Department of Food Science and Technology, Kaunas University of Technology, Radvilėnų˛ pl. 19, LT-50254 Kaunas, Lithuania; simona.razanaite@gmail.com (S.R.); laura.juriene@ktu.lt (L.J.); rita.kazernaviciute@ktu.lt (R.K.); michail.syrpas@ktu.lt (M.S.)

**Keywords:** rowanberry pomace, enzymatic treatment, ultrasound treatment, sugars, bread quality, antioxidant characteristics

## Abstract

Novel ingredients from rowanberry pomace were developed for French-type bread applications via supercritical CO_2_ extraction and the enzymatic and ultrasound treatment of the defatted residue (DFR), which contained 6.367% of proteins, 8.36% of soluble, and 43.04% insoluble fiber. Proteolytic enzymes from *Bacillus licheniformis* and *Aspergillus oryzae*, and cellulolytic enzyme mixtures Viscozyme L and Celuclast, were used to increase the soluble fraction. Treating DFR with enzymes generated significant amounts of soluble substances containing oligosaccharides, fructose, and glucose, with Viscozyme L being more effective than proteases. Tri-, and tetrapeptides, chlorogenic acids, and dihydroxy coumarins were also present in the soluble extracts of fermented DFR. The antioxidant characteristics of treated DFR were evaluated by the in vitro assays. Substitution of >5% of wheat flour with untreated DFR significantly reduced bread volume and crumb porosity; however, these adverse effects were mitigated by using fermented DFR. The highest bread volume (1845 cm^3^) and porosity (78.38%) were observed in bread containing 5% pomace that underwent enzymatic hydrolysis and ultrasound treatment. The substitution of flour with DFR significantly increased the antioxidant characteristics of bread samples and the substances generated during the in vitro digestion. It may be concluded that rowanberry pomace ingredients may improve bread nutritional quality and assist in the sustainable use of fruit processing by-products.

## 1. Introduction

Bread and bakery products are staple foods worldwide, with a $227.45 billion global market in 2024 and a compound annual growth rate (CAGR) of 5.3% [1]. Since it is a product consumed by a large portion of the population, regardless of age, it is ideally suited for enrichment and public marketing [2]. For instance, on average, Europeans consume 50 kg of bread per person per year or approximately 137 g per day [3]. Consequently, baked goods encompass a vast array of foods, with their fortification aimed particularly at enhancing health-beneficial properties and substituting the primary raw material, cereal flour, with alternative ingredients. Clinical trials have shown health benefits from consuming bread fortified with vitamins, minerals, proteins, fiber, and polyphenolic compounds [4]. Numerous unconventional raw materials have been used to improve the nutritional value of breads [5]. In line with sustainability and waste-reduction goals, food industry by-products have gained particular interest as functional ingredients in bakery products [6]. Improvement in the nutritional quality of baked goods with novel ingredients from fruit and vegetable by-products has also been widely studied [7]. Due to the positive influence of fibers and other health-beneficial compounds present in fruit and vegetable by-products, their use in baked goods containing glycemic carbohydrates, such as non-resistant starch, is ideal for bread. However, the use of fruit by-products in baked goods may reduce acceptability, mainly due to a lack of understanding of their physical structure and composition, as well as their effects on quality [7]. Birch and Bonwick [8] reviewed the functional, sensory, physical, and health-beneficial properties of bakery products; in many cases, reformulating bread by incorporating a single ingredient led to adverse effects, e.g., on the sensory and other characteristics of the final products.

Pressing of small fruit (commonly called berries) juice generates 10–35% of by-products—pomace, which contain various valuable nutrients [9]. These wastes are attractive due to their chemical composition, which includes polysaccharides, phenolic compounds, other phytochemicals, dietary fibers, natural antioxidants, and other compounds with positive health effects. It has been scientifically proven that the content of some bioactive substances in pomace can be higher than in fruit juice; therefore, one of the most promising ways to utilize berry by-products is their conversion into high-value-added natural ingredients for the creation of new products with increased nutritional value [10,11].

Although there is a wealth of scientific research on various berry pomaces, greater interest in rowan berry pomace, its positive health effects, and potential application in the food production process has emerged more recently. Rowan (*Sorbus aucuparia* L.) is a widespread deciduous tree species, most often grown as an ornamental plant, which produces small orange or pink fruits (6–9 mm in diameter) [12]. Rowan berries contain organic acids (ascorbic, malic, and citric), vitamins (C, E, K, and P), carotenoids, flavonoids, minerals (Zn, Fe, Mg, and Mn), carbohydrates, and other compounds with functional properties [12,13]. However, the studies on the use of rowan fruits and their ingredients in foods are relatively scarce. Borczak et al. [14] reported that adding 5% lyophilised wild-grown fruits, including rowanberries, to bread increased its antioxidant activity and total polyphenol content. Meremäe et al. [15] evaluated the effects of adding 2% rowan fruit powder to minced pork and beef on microbial growth inhibition, polyphenolic profile, and antioxidative capacity. Since the residual amounts of bioactive compounds in berry pomace may be even larger than in the whole fruit or pressed juice [9,10], rowanberry pomace might also be a promising raw material for the production of novel ingredients [16,17,18,19,20]. For instance, Tańska et al. reported 42.94 mg/100 g of vitamin C and 16.74 mg/100 g of total phenolic compounds in rowanberry pomace; substituting 20% of the flour with the pomace in the cookie formulation increased the cookie’s radical-scavenging capacity by almost 4 times [21]. In our previous work, we incorporated 2% rowanberry pomace into meatballs [22]. To the best of our knowledge, studies on the enzymatic and ultrasonic processing of rowanberry pomace and on the use of the resulting ingredients in bread have not been reported previously.

This study aims to evaluate various processing and extraction methods for converting rowanberry pomace into novel ingredients and to apply these methods to increase the nutritional and biological value of bread products. For achieving this aim the objectives of this study were (i) to investigate the chemical composition and antioxidant indicators of rowanberry pomace; (ii) to isolate rowanberry pomace fractions using enzymatic and ultrasonic processing methods and to evaluate their properties and composition; (iii) to evaluate the influence of selected rowanberry pomace ingredients on the quality, composition and antioxidant activity of bread; and (iv) to investigate the release of antioxidants at different stages of the in vitro gastrointestinal bread digestion process.

## 2. Materials and Methods

### 2.1. Materials

Fresh pomace of rowan (*Sorbus aucuparia* L.) fruits was kindly donated by UAB “Įvairios sultys” (Josvainiai, Lithuania). It consisted of seeds, pulp (mesocarp and endocarp), and skin (exocarp). The pomace was freeze-dried within 5 h of pressing and milled in a Retsch ZM 200 cyclone mill (Haan, Germany) using a 0.5 mm sieve.

6-Hydroxy-2,5,7,8-tetramethylchromane-2-carboxylic acid (TROLOX, 97%), 2,2′-azobis(2-amidinopropane) dihydrochloride (AAPH), Folin & Ciocalteu’s phenol reagent (2M), gallic acid (99%), microcrystalline cellulose (20 μm), D-(+)-glucose (>99%), 3,5-dinitrosalicylic acid (98%), phenol (>99%), potassium sodium tartrate tetrahydrate (C_4_H_12_KNaO_10_, Rochelle salt, 99%) and Viscozyme L from *Aspergillus* sp. and cellulase Celluclast 1.5 L from *Trichoderma reesei* were from Novozymes A/S (Bagsværd, Denmark); eroteolytic enzymes from *Bacillus licheniformis* were from Megazyme (Wicklow, Ireland), from *Aspergillus oryzae* Sigma-Aldrich (Hamburg, Germany); sodium acetate (CH_3_COONa, >99%), citric acid (C_6_H_8_O_7_, 99%, anhydrous), sodium sulphite (Na_2_SO_3_. 98.5%, anhydrous) and sodium hydroxide (NaOH, 98%, pellets) were from Acros Organics (Geel, Belgium); fluorescein (FL) was from Fluka Analytical (Bornem, Belgium); 2,2′-azino-bis(3-ethylbenzothiazoline-6-sulphonic acid) diammonium salt (ABTS), potassium chloride (KCl), sodium chloride (NaCl), potassium persulfate (K_2_S_2_O_8_) and sodium hydrogen phosphate (Na_2_HPO_4_) were from Merck (Darmstadt, Germany); potassium hydrogen phosphate (KH_2_PO_4_) was from Jansen Chimica (Beerse, Belgium); and sodium carbonate (Na_2_CO_3_, 98%, anhydrous) was from RPL (Grauwmeen, Belgium), food-grade ethanol was from Stumbras (Kaunas, Lithuania), and carbon dioxide (CO_2_, 99.9%) was from AGA (Vilnius, Lithuania). All solvents were of analytical and HPLC grade containing the enumerated chemicals used.

### 2.2. Defatting of Pomace by Supercritical Fluid Extraction with CO_2_ (SFE-CO_2_)

**Figure 1:**
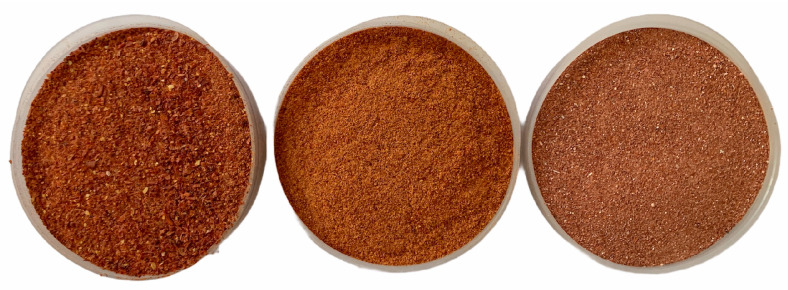
Dried rowanberry pomace (**left**), milled using a 0.5 mm sieve (**middle**), and after SFE-CO_2_ (**right**).

The pomace (5750 g) was defatted by SFE-CO_2_ in a 10 L pilot-scale extractor (Applied Separations, Allentown, PA, USA) at the previously optimized parameters: 45 MPa pressure, 50 °C temperature, and constant CO_2_ flow rate of 2.5 L/min [23]. The extract was collected and weighed to determine its yield. Defatted pomace powder was stored in tightly closed containers in a dry, dark room at a temperature not exceeding 20 °C. The products are shown in Figure 1.

### 2.3. Determination of Proximate Pomace Composition and Particle Size

The AOAC methods were adapted for the determination of proximate composition [24]. Briefly, the moisture content was determined using a Moisture Analyser MB64 60G (BEL Engineering s.r.l., Monza, Italy). The content of minerals (ash) was determined after incineration of organic substances at 600–650 °C for 2 h. Protein content was determined by the Kjeldahl method. Briefly, 1 g of ground pomace was mineralised in an InKjel P apparatus, then distilled in a Behr S4 apparatus using program no. 2, and finally the solutions were diluted with 0.1 M HCl. The fat content was determined by the Soxhlet method in a Behr R 604 apparatus (all from Behr-Labor Behrtest, Düsseldorf, Germany) using hexane.

The amount of fiber was determined using the enzymatic method in a Fibertec 1023 (Foss, Hilleroed, Denmark) apparatus, reference method AOAC 991.43 [24] using a Total Dietary Fiber Assay Kit (Megazyme, Bray, Ireland). The process is based on the enzymatic digestion of samples with thermally stable α-amylase, protease, and amyloglucosidase.

A Malvern Mastersizer 2000 (Malvern Instruments Ltd., Malvern, Worcestershire, UK), connected to a Hydro 2000S dispersion system, was used to determine the particle size distribution. Briefly, the pomace was dispersed in distilled water at 1330 rpm, and the angular changes in the intensity of scattered light as the laser beam passed through the solid particle sample were measured. The data were used to calculate the volume-weighted mean diameter (D_[4,3]_), the surface-weighted mean diameter (D_[3,2]_), and the span factor, which indicates the uniformity of the particle size distribution.

### 2.4. Fractionation of Pomace

#### 2.4.1. Protein Fractionation

Protein fractionation was performed using a classical Osborne procedure. The method is based on the different solubility properties of the protein fractions: albumins are soluble in water and dilute buffers; globulins are soluble in salt solutions; gliadins are soluble in 70–90% ethanol; and glutelins are soluble in dilute acids or alkali solutions. A detailed description of the procedure is presented in the Supplementary Materials.

#### 2.4.2. Extraction of Soluble Substances by Treating with Enzymes and Ultrasound

To evaluate the extraction efficiency of soluble compounds, defatted pomace was treated under different conditions using alkalis, ultrasound (US), and proteolytic and cellulolytic enzymes. The extraction conditions were selected based on the methods described by Görgüç et al. [25] with minor modifications. A detailed description of the procedure is presented in the Supplementary Materials, Section S1.

The content of fiber in the pomace in the enzyme and/or ultrasound-treated products was determined in the Fibertec™ 1023 apparatus as described in the Supplementary Materials. The insoluble fraction remaining after the treatments was subjected to enzymatic hydrolysis using Viscozyme L and Celluclast. The insoluble fraction was mixed with distilled water (1:10) and incubated with 0.1 mL/g enzyme for 4 and 7 h at 50 °C; the mixture was then heated to 95 °C for 20 min to inactivate the enzymes, cooled to 30 °C, filtered, and centrifuged in a Velocity 18 R (Dynamica Scientific, Livingston, UK) for 30 min at 4800 rpm. The separated aqueous fraction was lyophilized in a Maxi Dry Lyo (Jouan Nordic A/S, Allerød, Denmark) at 0.5 mbar and −40 °C for ~24 h, and the yield was calculated. The extracts were stored in a dry, cool place until further analysis.

### 2.5. Determination of Carbohydrate Composition by (HPLC)

After enzymatic hydrolysis of the pomace and its fibers, the resulting extracts were dissolved in ultrapure water (Millipore, Bedford, MA, USA) at a concentration of 10 mg/mL. Chromatographic analysis was performed using a Thermo Scientific Ultimate 3000 HPLC system equipped with a RefractoMax 521 refractive index detector, Chromeleon 7 software (Thermo Fisher Scientific, Waltham, MA, USA), and SUGAR KS-801 and KS-802 chromatographic columns (8.0 × 300 mm, Shodex, Tokyo, Japan). The analysis conditions were as follows: isocratic, mobile phase purified water at a flow rate of 0.5 mL/min, injection volume 10 µL, separation run time 45 min, column temperature 80 °C, and the detector temperature 55 °C. The compounds were identified by their elution times relative to the standards. External calibration curves were constructed for sucrose, glucose, fructose, kestose, nystose, and fructofuranosylnystose (0–2.0 mg/mL, R2 ≥ 0.9999). The concentrations of the identified compounds were calculated from the peak areas or heights and expressed as mg/g DM.

### 2.6. Preliminary Screening of the Composition of Hydrolyzed Pomace by UPLC-QTOF

The lyophilised extracts obtained after enzymatic hydrolysis of pomace were dissolved in ultra-pure water at a concentration of 1 mg/mL and analyzed on a Waters AQCUITY ultra performance liquid chromatography system (UPLC, Waters Corp., Milford, MA, USA) equipped with a quadrupole time-of-flight mass spectrometer (maXis 4G QTOF) and HyStar 3.2 SR2 software (Bruker Daltonics, Bremen, Germany). The compounds were separated on an Acquity BEH C18 column (1.7 µm, 50 × 2.1 mm) with formic acid solution (A) and methanol (B) at the flow rate of 0.4 mL/min and the following linear gradient: B from 0 to 100% (0–9 min); B 100% (9–10 min); B from 100 to 0% (10–12 min). The column was equilibrated for 2 min before each run. The QTOF-MS was set to positive ionization mode, with a voltage of +4000 V. Nitrogen was used as the sparging and drying gas (2.5 bar pressure) at 200 °C, with a flow rate of 10 L/min. Tentative peak identification was performed by comparing MS spectra and, for some analytes, retention times with data from the literature and the METLIN database.

### 2.7. Production and Evaluation of Bread with Pomace Products

#### 2.7.1. Production of Bread

A simple French bread recipe was chosen for bread production, which was slightly adjusted for each batch based on the amount of added pomace (Table 1). The bread was made in an electric breadmaker, the Russell Hobbs Classics 18036-56 (Oldham, UK). The ingredients were loaded into the baking pan in the following order: (1) room temperature water; (2) table salt (SE Artyomsalt, The Ukraine), (3) low-extraction premium wheat flour type 550D, consisting of 1.3 g fat, 73.6 g carbohydrates, 0.50 g sugars, 10.4 g protein (Malsena, Panevėžys, Lithuania), and (4) dry yeast (Dr. Oetker, Vilnius, Lithuania).

In the recipes with pomace, 5, 7.5, and 10% of the flour was substituted with the defatted pomace. The amount of water was slightly increased depending on the amount of pomace, since pomace absorbs more water than wheat flour. Baking parameters were selected to achieve the largest bread size and an average level of browning. Mixing, forming the dough, proving, and baking were performed automatically in the bread maker for 3 h and 41 min. The bread was removed from the bread maker, left to cool at room temperature, and the quality characteristics were evaluated after approximately 24 h.

#### 2.7.2. Application of Ultrasound (US) and Enzymatic Hydrolysis in Bread Production

To assess the effect of enzyme- and US-treated pomace on the quality of bread, pomace was processed with an ultrasonic probe and/or Viscozyme L (the details in Supplementary Materials) before being mixed into the bread recipe. For this purpose, 18 g (5%), 27 g (7.5%), and 36 g (10%) of pomace were mixed with 180, 185, and 190 mL of water, respectively, and ultrasonicated for 20 min at 200 W with a maximum temperature of 50 °C. Afterwards, 1.08 mL of Viscozyme L (0.06 mL/g pomace) was added, and the mixture was incubated at 40 °C for 4 h. After enzymatic hydrolysis, the product was dosed into the dough, with an additional 30 mL of water added according to the elaborated recipe (Table 1), while all baking parameters remained identical.

#### 2.7.3. Determination of Bread Volume, Crumb Porosity, and Moisture

The volume of bread products is estimated from the amount of millet grains displaced by the baked product, measured with a measuring cylinder and expressed in cm^3^. The porosity of the bread crumbs was assessed according to the standard method LST 1442:1996/P:2020 [26], which is based on the ratio of the bread crumb pores to the total crumb volume. A detailed description is presented in the Supplementary Materials.

#### 2.7.4. In Vitro Gastrointestinal Digestion of Bread by the Global Antioxidant Response (GAR) Method

After assessing the bread’s quality indicators, the bread crumbs were crushed by hand and transferred to a 60 °C airflow dryer Food & Jerky Dehydrator StandArt (Witeg Labortechnik GmbH, Wertheim, Germany) for 8–12 h. The dried bread was ground in a laboratory cyclone mill with a mesh size of 0.5 mm and 0.2 mm. After grinding, the resulting material was stored in sealed glass containers at room temperature until further research. In vitro gastrointestinal digestion of bread was performed using the method of Miller et al. [27], modified to include an oral step (see Supplementary Materials for detailed description).

### 2.8. Determination of Antioxidant Properties in Extracts and Solid Fractions

The antioxidant potential of various solid fractions and extracts was assessed by the ABTS^•+^ scavenging capacity (decolorization) [28], total phenolic content (TPC) [29], and oxygen radical absorbance capacity (ORAC) [30] methods. The extracts were dissolved in distilled water to obtain concentrations of 0.5, 0.25, 0.125, 0.0625, 0.03125, and 0.015625 mL/mL. The QUENCHER procedure was applied for the solids [31] with slight modifications. Dried and ground samples were mixed with microcrystalline cellulose (20 μm) in a Bio Vortex V1 plus (BioSan, Riga, Latvia), and six dilutions were prepared to obtain concentrations of 0.5, 0.1, 0.05, 0.02, 0.01, and 0.005 mg/mg. A detailed description is presented in the Supplementary Materials.

### 2.9. Statistical Data Analysis

The results obtained during the study were handled in Microsoft Excel. The data are presented as the mean values ± standard deviation (SD, %) from three replicate experiments. Differences among samples differing in a single characteristic were assessed using a one-way ANOVA, followed by Tukey’s post hoc analysis. The selected statistical significance level was *p* < 0.05. The data were considered statistically reliable if they were below the specified value and unreliable if they exceeded it.

## 3. Results and Discussion

### 3.1. Proximate Composition of Rowanberry Pomace

The proximate composition of rowanberry pomace was evaluated before and after SFE-CO_2_ (Table 2). The results show that the percentage content of proteins, minerals, and soluble fiber after SFE-CO_2_ increased by 6.5%, 11.3%, and 27.4%, respectively, while insoluble fiber content decreased slightly. The CO_2_ used for SFE is a non-polar solvent, which effectively extracts lipophilic compounds; therefore, after removal of the significant part of the oil, the percentage content of other, CO_2_-insoluble constituents increases. Moreover, treatment of pomace at high pressure during SFE-CO_2_ may alter its structural properties, thereby increasing soluble fiber content. Some differences in the appearance of pomace before and after SFE-CO_2_ may be observed in Figure 1.

Reißner et al. [32] reported a slightly lower fat content (3.97%), a higher protein content (7.09%), and a higher insoluble dietary fiber content (59.5%) in rowanberry pomace. Several factors may account for the differences observed in our data, including fruit cultivar, harvest time, climatic conditions, and soil composition. It may be noted that 4.27 g/100 g of lipids was recovered by SFE-CO_2_, which is 10% lower compared with the Soxhlet method; however, in agreement with the previously reported results by Bobinaitė et al. [17], who recovered 4.8% of lipids from the dried rowanberry pomace. The size of particles in the ground materials is essential both in the extraction and application of rowanberry pomace in bread formula; therefore, the particles were evaluated before and after SFE-CO_2_ (Table 2). SFE-CO_2_ does not mechanically reduce particle size during the process. However, it can indirectly influence particle integrity by removing lipophilic substances that may act as structural binders within the pomace matrix. Lipids help maintain cohesion between cell fragments; thus, their removal weakens this structure, making particles more brittle and prone to fragmentation during subsequent handling steps such as sieving [20]. The observed reduction in particle size after SFE-CO_2_ is likely due to this loss of structural integrity combined with mechanical forces applied post-extraction, rather than the extraction process itself.

### 3.2. Extraction of Soluble Substances from Rowanberry Pomace

#### 3.2.1. Protein Fractionation by the Osborne Method

The composition of fruit proteins and their morphological properties are significantly different from cereal proteins. Since gluten, as a complex of wheat proteins primarily consisting of gliadin and glutenin, is significant in the formation of bread dough structure and particularly its elasticity, it was of interest to determine rowanberry protein composition by using a classical Osborne method. The yields of different fractions of rowanberry proteins were as follows: albumin, 49.89 ± 0.26%; globulin, 1.28 ± 0.01%; prolamin, 16.30 ± 0.13%; gliadin, 69.40 ± 2.91%. For comparison, wheat proteins on average consist of 5% albumin, 10% globulin, 45% prolamin, and 40% glutelin. The available scientific information on small fruit proteins is very scarce. A comprehensive evaluation of blackberry seed protein composition and properties was recently reported by Wang et al. [33]. The study demonstrated that glutelin exhibited strong water-holding, foaming, and emulsification capacities, while globulin and albumin showed strong oil-holding capacity and thermal stability. Gliadin and glutenin are subunits of gluten, which, together with water, form a protein network of bread dough. The primary function of this network is to provide the dough with viscosity and elasticity. Therefore, it may be expected that, due to the high content of gliadins, the use of rowanberry pomace in the production of bread may be acceptable in terms of its effect on the texture of the final product.

#### 3.2.2. Extraction of Soluble Substances from Rowanberry Pomace by Different Methods

To evaluate the efficiency of soluble substance extraction, the study used different methods, with some samples additionally exposed to US. The enzyme concentrations for the studies were selected based on previous scientific publications. From the presented results (Table 3), it can be seen that the highest yield (48.94 ± 0.29%) was obtained for the sample that underwent enzymatic hydrolysis with the Viscozyme L and was additionally treated with US. In comparison, the lowest yield (21.72 ± 0.70%) was obtained by alkaline extraction. Alkaline extraction has been widely used for the recovery of proteins; however, higher yields can be obtained only at more alkaline conditions, which can degrade protein nutritional value [34]. At optimal parameters, Baca-Bocanegra et al. [35] recovered 59.13 g protein/100 g of alkaline protein isolate of the defatted grape seed meal; whereas Salem et al. [36] from the defatted and dephenolized seeds of two grape varieties under alkaline conditions recovered 3.90% and 4.11% of protein extract with 63.95 and 57.93 mg/g DM. However, grape seeds contained a remarkably higher protein content than the rowanberry pomace used in our study. The ultrasound treatment increased the yield of soluble substances by 13% compared with alkaline extraction. The use of US in the extraction of protein compounds is based on elastic mechanical vibration waves, which accelerate mass transfer; as a result, the cavitation effect more efficiently releases proteins from the parenchyma cell wall [37]. Ultrasound-assisted extraction is a green technology that has been increasingly used for the recovery of proteins from plant-based materials, mainly as a pre-treatment method that disrupts the cell–matrix and thereby improves extractability. Additionally, US has also been applied to modify the physical, structural, and functional properties of protein-based ingredients [38].

Viscozyme L, an effective multicomponent carbohydrase comprising xylanase, arabinanase, β-glucanase, hemicellulase, and cellulase, has been widely used in enzyme-assisted extraction to hydrolyze plant cell wall polysaccharides, primarily to extract polyphenols and fermentable sugars [39]. Hydrolyzed carbohydrates release intracellular compounds, such as proteins and sugars. Guan and Yao [40] reported that enzyme-assisted extraction of oat bran with Viscozyme L under optimal conditions recovered significantly more protein (56.2%) than the alkaline method (14.76%).

Comparing the results obtained by enzymatic hydrolysis with commercial proteases from *A. oryzae* and *B. licheniformis*, the differences between the experiments were not significant, and the yields ranged from 38.23 to 43.23% (Table 3). The increase in concentration of protease from *B. licheniformis* from 0.01 to 0.02 mL/g pomace increased the yield of soluble substances by 11% (no US) and 6% (with US). In contrast, a higher concentration of protease from *A. oryzae* did not significantly affect extract yield. However, this yield was still lower than that obtained with Viscozyme L. Proteases are enzymes that catalyze the breakdown of only proteins [41]. In contrast, Viscozyme L has a much broader spectrum of action, particularly in materials containing low protein and high carbohydrate levels. US pre-treatment, in case of using proteases, slightly and in some cases significantly increased extract yields.

After enzymatic hydrolysis, the solid fraction was dried, and the fiber content in the samples was estimated (Table 3). The highest fiber content (78.75 ± 1.58%) was determined in the sample treated with protease from *B. licheniformis* (0.01 mL/g). After US, the yield decreases by 13.7%. A slightly lower content was observed in the *A. oryzae*-treated sample (77.10 ± 0.45%), but US reduced the yield by 6.6%. The more soluble fractions are separated from the rowanberry pomace during enzymatic hydrolysis, the lower the sample’s fiber content remains. In this case, the yield of soluble compounds using the enzyme mixture Viscozyme L and additional US treatment was the highest (48.94 ± 0.29%). Still, the fiber content of the centrifuged product from this sample was the lowest (67.38 ± 0.20%). In addition, the slightest negative change in US fiber content (3.8%) was also found in the sample with the Viscozyme L. Based on the obtained data, it can be concluded that the use of US during enzymatic hydrolysis reduces the amount of fiber in the solid fraction. Ultrasound treatment alone can reduce dietary fiber content by disrupting cell wall structures through cavitation, which generates intense shear forces and microjets. These forces could fragment insoluble polysaccharides in rowanberry pomace into smaller oligosaccharides or monosaccharides, thereby lowering the fraction classified as dietary fiber [42]. When combined with Viscozyme, ultrasound improves enzymatic accessibility, accelerating fiber hydrolysis and solubilization. Similarly, pairing ultrasound with Alcalase promotes protein breakdown, further weakening the structural matrix and indirectly facilitating fiber degradation [43].

### 3.3. Antioxidant Activity in Lyophilized Soluble Substances

**Figure 2:**
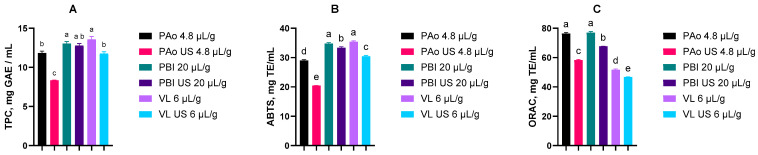
Different figures indicate different assays. Total Phenolic Content (TPC, (**A**)), ABTS^•+^-scavenging (**B**), and ORAC (**C**) values of rowanberry pomace lyophilizates after enzymatic hydrolysis: PAo, protease from *A. oryzae*; PBl—protease from *B. licheniformis*; VL—Viscozyme L; US—ultrasound. Values are expressed as mean ± standard deviation, *n* = 3. Different lowercase superscript characters indicate significant differences between values (one-way ANOVA followed by Tukey’s post hoc test, *p* < 0.05).

Antioxidant characteristics of soluble substances of rowanberry pomace after the enzymatic treatment with or without the use of US are summarized in Figure 2. The enzyme concentration was selected based on the highest yield (Table 3). The TPC values were in the range from 8.34 ± 0.05 mg GAE/mL (the sample treated with protease from *A. oryzae* and US) to 13.58 ± 0.32 mg GAE/mL (the sample that underwent enzymatic hydrolysis with Viscozyme L). Similarly, the lowest and highest values of ABTS^•+^-scavenging capacity were determined in the same samples and ranged from 20.45 ± 0.05 to 35.43 ± 0.47 mg TE/mL. The distribution of ORAC values ranged from 46.7 ± 0.19 mg TE/mL in the sample treated with Viscozyme L and US to 77.02 ± 1.14 mg TE/mL in the sample hydrolyzed with *B. licheniformis*.

Different mechanisms of the applied assays may explain these findings: the Folin–Ciocalteu and ABTS^•+^-scavenging methods are based on single-electron transfer, whereas the ORAC method measures peroxyl radicals and is therefore more closely related to processes occurring in biological systems. In general, the differences in the effects of the applied enzymes on the values obtained from single-electron transfer-based assays were not remarkable. In contrast, ORAC values were significantly higher in the samples treated with proteolytic enzymes than in the samples hydrolyzed with Viscozyme L. At first sight, it might look surprising that the combined treatment with enzymes and US resulted in lower values in all assays. One possible explanation is that the US increased the yield of soluble substances, thereby diluting antioxidative constituents with more neutral ones. Another group of essential factors concerns the effects of enzymatic hydrolysis. In plant materials, which contain low levels of protein, TPC and other antioxidant properties are associated with polyphenolic constituents. However, the applied chemical antioxidant capacity assay methods are not selective to phenolic antioxidants. During protein hydrolysis, numerous products, such as peptides and amino acids, are formed and may participate in redox reactions similar to those of phenolic antioxidants [44]. Therefore, in such cases, antioxidant properties may be considered only as the indicative characteristics. Sarv et al. [19] reported antioxidant properties of fruits, juice, and pomace of 17 rowan cultivars from Estonia: TPC values of pomace varied from 15.97 to 44.68 mg GAE/g dw whereas ABTS^•+^-scavenging capacity and ORAC were in the range of 45–146 mg TE/g and 11–37.7 mg TE/g dw, respectively. The results of preliminary studies on hydrolysates, as presented in the following sections, may partially support this hypothesis.

### 3.4. Preliminary Screening of the Composition of Soluble Extracts

Comprehensive qualitative and quantitative analysis of the enzyme-treated pomace was beyond the scope of this study. Since the samples of rowanberry pomace were treated with proteolytic enzymes, preliminary screening of solubilized fractions was performed by UPLC-Q/TOF. It was expected that proteases might produce smaller-molecular-weight peptides. For this purpose, two samples with the highest yields of soluble substances were selected: with protease from *A. oryzae* (0.0048 mL/g) and *B. licheniformis* (0.02 mL/g). The results, which are presented in Table 4, show that the main constituents detected by the applied method are tri- and tetrapeptides, organic and phenolic acids, and hydroxycoumarins. It was not possible to determine the exact peptide composition because various peptide combinations yield similar *m*/*z* values. The identification of precise isomers of hydroxycoumarins and caffeoylquinic (chlorogenic) acids, which depend on the position of the hydroxy group and the site of the ester link between caffeic and quinic acids, respectively, was also not among the objectives of this study.

Some differences in the composition of soluble extracts obtained by using different enzymes may be observed. The differences in peptide composition may depend on the proteases used. Scientific information on rowanberry amino acids is very scarce. Sergunova et al. [45] reported threonine and proline as dominant amino acids in the dried rowanberries after hydrolysis with hydrochloric acid.

Quantitative data may be evaluated preliminarily based on peak *m*/*z* intensities. For instance, a higher concentration of caffeoylquinic acid isomers was observed in the sample treated with *B. licheniformis* proteinase. In comparison, a lower concentration was observed in the extract obtained with proteinase from *A. oryzae*. Chlorogenic acid is one of the main phenolic acids in rowanberries [46]. Bobinaitė et al. [18] reported 3.4 mg/g of chlorogenic acid in the aqueous extract of rowanberry pomace. In our study, mevalonic and sorbic acids were also found in the lyophilized pomace. Based on the *m*/*z* intensity, a higher amount of mevalonic acid was found in the sample hydrolyzed with proteinase from *B. licheniformis*, and sorbic acid in the sample with proteinase from *A. oryzae*. However, a more comprehensive evaluation of the concentrations would be required to enable precise quantification; therefore, our findings should be considered preliminary.

On the other hand, differences in the organic acids and other metabolites after protease treatment may be due to differences in the mode of enzyme activity, which can release molecules bound to the matrix. For instance, protease from *B. licheniformis*, the commercial form of endopeptidase subtilisin, is a cross-linked enzyme aggregate. In comparison, protease from *A. oryzae* is a mix of endo- and exo-peptidases. It is interesting to note that the common name of sorbic acid is derived from the *Sorbus* genus name because it was isolated in 1859 by distillation of rowanberry oil by A. W. von Hofmann [47]. Sorbic acid is a well-known antimicrobial compound and is approved as a preservative in many countries. Mevalonic acid is also common to many plants as an intermediate compound in the biosynthesis of terpenoids. Based on the preliminary evaluation of the composition of soluble substances, it may be expected that, in such a way, processed rowanberry pomace will acquire enhanced bioactive properties compared with the untreated pomace.

### 3.5. Sugar Composition in the Hydrolyzed Pomace

Sugars are essential ingredients in baking. Therefore, after enzyme treatment of rowanberry pomace, the sugar composition of the lyophilized products was evaluated (Table 5). The highest content of mono- and disaccharides (392.56 ± 3.70 mg/g dw) was determined in the soluble substances obtained after hydrolysis with Viscozyme L (12.0% and 14.2% more than in the samples treated with proteases from *B. lichniformis* and *A. oryzae*, respectively), mainly due to the content of the main fermentable sugars saccharose, fructose, and glucose, fructose being the most abundant sugar. To the best of our knowledge, data on sugars in rowanberry pomace is not available. Zymone et al. [46] reported data on 20 *Sorbus* species and cultivars: glucose and fructose were the main sugars, present at concentrations of 53.37–221.07 and 18.45–187.28 mg/g dw, respectively. Consequently, the soluble fractions of the enzyme-treated pomace contained comparably high amounts of sugars. Gullon et al. [48] reported fructose as the predominant monosaccharide in the hydrolyzed apple pomace extract. The reduction in oligosaccharides in the Viscozyme L-treated samples can be attributed to the enzyme’s ability to hydrolyze complex polysaccharides and intermediate oligosaccharides into smaller monosaccharides, thus lowering their detectable levels. The total oligosaccharide content includes four groups of compounds with a polymerization degree (DP) of 3, 4, 5–6, and more than 7. The content of galacturonic acid, the main monomer of pectins, was also significantly higher in samples hydrolyzed with Viscozyme L; this may be explained by the pectinase activity of the enzyme mixture, which can degrade the cell wall more effectively than the proteases.

**Table 5:** Sugars in the freeze-dried soluble pomace substances after enzymatic hydrolysis (not treated with ultrasound), mg/g dw. DP—degree of polymerization.

Component	Protease		Viscozyme L, 0.06 mL/g
	*A. oryzae*, 0.0072 mL/g	*B. lichniformis*, 0.01 mL/g	
DP > 7	10.31 ± 0.07 ^b^	10.18 ± 0.12 ^b^	9.04 ± 0.14 ^a^
Galacturonic acid	111.9 ± 3.1 ^a^	111.5 ± 2.7 ^a^	148.2 ± 1.8 ^b^
DP 5–6	-	2.69 ± 0.10	-
Nystose (DP 4)	1.11 ± 0.08 ^a^	2.69 ± 0.07 ^b^	1.13 ± 0.03 ^a^
Kestose (DP 3)	2.95 ± 0.20 ^b^	5.24 ± 0.01 ^c^	1.86 ± 0.06 ^a^
Sucrose (DP 2)	7.04 ± 0.05 ^a^	8.91 ± 0.40 ^b^	11.76 ± 0.53 ^c^
Glucose	54.52 ± 0.67 ^a^	52.28 ± 0.85 ^a^	58.04 ± 0.42 ^b^
Fructose	158.2 ± 0.6 ^a^	157.3 ± 2.1 ^a^	167.5 ± 1.1 ^b^
Sugar alcohols	8.21 ± 0.36 ^b^	10.36 ± 0.64 ^c^	7.00 ± 0.08 ^a^
Total oligosaccharides	14.38 ± 0.35 ^b^	20.80 ± 0.08 ^c^	12.03 ± 0.05 ^a^
Total mono- and disaccharides	339.9 ± 4.9 ^a^	340.4 ± 0.8 ^a^	392.6 ± 3.7 ^b^
Total	354.3 ± 5.2 ^a^	361.2 ± 0.9 ^a^	404.6 ± 3.7 ^b^

Values are expressed as mean ± standard deviation, *n* = 3. Different lowercase superscript characters indicate significant differences between values within rows (one-way ANOVA followed by Tukey’s post hoc test, *p* < 0.05). In addition, the insoluble fraction remaining after protease hydrolysis was further hydrolyzed with Celluclast and Viscozyme L for 4 h. The hydrolysis of the *B. licheniformis* proteinase-treated residue resulted in the generation of 19.43 ± 0.11% and 27.70 ± 0.19%; A. *oryzae* proteinase-treated, 13.97 ± 0.06% and 18.65 ± 0.54% of soluble substances, respectively (Table 6). Increasing the treatment time to 7 h did not yield positive results; therefore, the soluble fractions obtained from the hydrolysis of the *B. licheniformis* proteinase-treated solid residue were used for saccharide analysis. It may be mentioned that the highest content of insoluble substances (78.75 ± 1.58%) was determined for the sample treated with 0.01 mL/g of proteinase from *B. licheniformis* without US (Table 3).

**Table 6:** Sugars in the freeze-dried solid pomace fractions (in mg/g dw) after enzymatic hydrolysis and dietary fiber hydrolysis with protease from *B. lichniformis* (0.01 mL/g).

Constituents	Celluclast		Viscozyme L	
	no US	US	no US	US
DP > 7	4.20 ± 0.19 ^a^	5.25 ± 0.02 ^b^	4.08 ± 0.13 ^a^	18.95 ± 0.38 ^c^
Galacturonic acid	40.55 ± 1.83 ^a^	52.82 ± 2.07 ^b^	68.97 ± 1.76 ^c^	76.30 ± 1.64 ^d^
DP 5–6	2.89 ± 0.12 ^a^	5.09 ± 0.13 ^b^	-	8.96 ± 0.24 ^c^
Nystose (DP 4)	-	-	-	1.79 ± 0.04
Kestose (DP 3)	2.32 ± 0.09 ^b^	0.66 ± 0.04 ^a^	2.30 ± 0.07 ^b^	3.54 ± 0.14 ^c^
Sucrose (DP 2)	5.40 ± 0.14 ^a^	5.25 ± 0.20 ^a^	11.96 ± 0.48 ^c^	9.36 ± 0.05 ^b^
Glucose	15.51 ± 0.07 ^a^	18.18 ± 0.42 ^b^	40.68 ± 0.02 ^d^	27.55 ± 0.49 ^c^
Fructose	83.11 ± 0.53 ^d^	74.27 ± 0.20 ^b^	80.36 ± 0.03 ^c^	67.15 ± 1.68 ^a^
Sugar alcohols	1.62 ± 0.06 ^a^	3.61 ± 0.01 ^b^	9.55 ± 0.20 ^c^	9.20 ± 0.10 ^c^
Total oligosaccharides	9.41 ± 0.01 ^b^	10.99 ± 0.15 ^c^	6.38 ± 0.20 ^a^	33.24 ± 0.04 ^d^
Total mono- and disaccharides	146.2 ± 1.4 ^a^	154.1 ± 2.9 ^b^	211.5 ± 2.4 ^d^	189.6 ± 3.9 ^c^
Total saccharides	155.6 ± 1.4 ^a^	165.1 ± 3.0 ^b^	217.9 ± 2.6 ^c^	222.8 ± 3.8 ^c^

Values are expressed as mean ± standard deviation, *n* = 3. Different lowercase superscript characters indicate significant differences between values within rows (one-way ANOVA followed by Tukey’s post hoc test, *p* < 0.05).

### 3.6. Use of Pomace Ingredients in Bread

#### 3.6.1. Influence of Pomace Ingredients on the Quality Indicators of Bread

To assess the influence of pomace ingredients on bread quality, part of the wheat flour (5, 7.5, and 10%) was replaced with defatted SFE-CO_2_ rowanberry pomace, which was untreated or treated with the enzyme Viscozyme L (0.06 mL/g pomace) and US (Table 7). The untreated defatted pomace reduced the volume and porosity of bread, particularly at higher addition levels (above 5%). For instance, when 10% pomace was used, the bread volume and porosity decreased by 26% and 14%, respectively. Similar effects were previously reported for grape pomace used at 5% and 10% in bread [49]. Replacing 10% of wheat flour with blackcurrant pomace reduced bread volume by 10.8% [50]. The high fiber content in berry pomace may adversely affect dough/batter viscosity and the texture and structure of the final bakery products [51]. The interactions of berry pomace fiber with the dough gluten matrix through hydrogen bonds increase structural strength, and the gluten becomes less developed. Therefore, it was suggested that, due to the disruption of the gluten film by fiber incorporation and its influence on dough properties, blackcurrant pomace at up to 10% to produce bread with satisfying characteristics [52].

**Figure 3:**
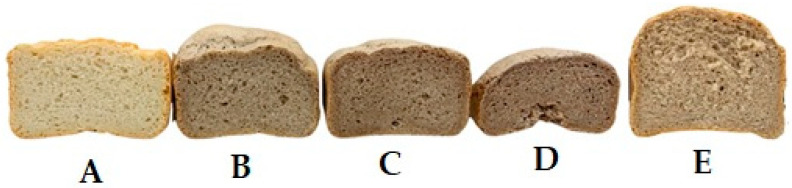
Effect of rowanberry pomace ingredients on bread: (**A**) control; (**B**) with 5%, (**C**) with 7.5%, (**D**) with 10% Viscozyme L-treated pomace; (**E**) with 5% US and Viscozyme-treated pomace.

In our study, when rowanberry pomace was treated with Viscozyme L, its adverse effects on bread quality were significantly reduced; moreover, when 5% pomace was added, the bread volume increased by 4.6%. Even better results were obtained when the pomace was treated with US and Viscozyme L: in this case, the addition of 5% (Figure 3) increased the volume and the porosity compared with the control sample by 17.7 and 6.9%, respectively, while for 7.5% addition, these indicators did not differ from the control bread. These improvements can be explained by the fact that the polysaccharides in the US-treated pomace become more accessible to enzymatic hydrolysis, which produces more yeast-friendly carbohydrates during dough fermentation. Increasing the pomace content in the dough increases water absorption, so recipes must be adjusted accordingly [52,53]. However, as the pomace content increases, the bread hardness may also increase, as it was shown with apple, where its percentage in blends increased up to 11% [53]. On the contrary, the hardness of Barbari bread produced with up to 7% of tomato pomace powder was less than that of other samples; the effect was related to bread moisture [54]. The increased water absorption is due to the higher dietary fiber content. The hydroxyl groups in dietary fiber interact with water molecules via hydrogen bonds [55]. In addition, enzymatic hydrolysis leads to a larger volume of the baked goods, and increased crumb porosity, and the additional use of US further increases these indicators [56].

#### 3.6.2. Antioxidant Properties of Rowanberry Pomace and Bread

Antioxidant characteristics were evaluated in rowanberry pomace before and after SFE-CO_2_, as well as in bread samples (Table 8). The antioxidant activity in pomace before and after SFE-CO_2_ does not differ significantly. The TPC values increased slightly, while ORAC and ABTS^•+^-scavenging capacity did not vary significantly. During SFE-CO_2_, lipophilic antioxidants are extracted, which may reduce antioxidant activity, while the concentration of phenolics, which are not soluble in supercritical CO_2_, may increase in the extraction residue.

Enzyme and ultrasound-assisted extractions may increase the antioxidant activity values of the extracts [57,58]. However, when using in vitro assays that rely on single-electron (SET) and/or hydrogen-atom (HAT) transfer, such an increase is not necessarily due to the release of phenolic antioxidants. Many other compounds belonging to different classes may participate in these SET/HAT reactions. However, the main reason for not performing these assays on the enzyme- and/or US-treated samples was that, rather than extracts, solid materials were added to the bread. In addition, it may be assumed that when adding the developed ingredients to bread, the most important results are obtained through gastrointestinal digestion (GID) simulation. In this case, we used both US-treated and enzyme-treated samples at the concentrations that yielded the best bread quality characteristics. Consequently, the results presented in the following subsection (Table 9) are more closely related to possible health benefits, specifically antioxidant properties.

A comprehensive scientific evaluation of the effects of rowanberry pomace ingredients on the sensory quality of bread was not conducted in our study. However, preliminary results, which are presented in Supplementary Materials (Section S6.4), as well as previously reported data on the effects and acceptability of bread produced with berry pomace ingredients, provide preliminary support for replacing wheat flour with rowanberry pomace without compromising bread quality [51,59,60,61,62,63].

#### 3.6.3. Evaluation of Antioxidant Activity During In Vitro Digestion of Samples

Antioxidant properties after in vitro digestion were evaluated in two bread samples. Bread without additives and bread with 5% fermented rowanberry pomace after SFE-CO_2_+US (Table 9) were selected, which showed the best quality indicators. The data presented show that the antioxidant activity of bread containing fermented rowanberry pomace is higher than that of the control sample. The most significant amounts of antioxidants are released in the gastric phase under the action of the enzyme pepsin. A somewhat smaller amount is determined in the intestinal phase under the action of pancreatic bile extract, and the smallest amount is released in the oral phase under the action of amylase solution.

## 4. Conclusions

Novel ingredients from rowanberry pomace were developed for French-type bread applications via supercritical CO_2_ extraction and the enzymatic and ultrasound treatment of the defatted residue (DFR), which contained 6.367% of proteins, 8.36% of soluble, 43.04% insoluble fiber, 2.56% minerals, and 36.20% other carbohydrates. Proteolytic enzymes from *B. licheniformis* and *A. oryzae*, and cellulolytic enzyme mixtures, Viscozyme L, and Celuclast significantly increased the soluble pomace fraction. Treating DFR with enzymes generated significant amounts of soluble substances containing oligosaccharides, fructose, and glucose, with Viscozyme L being more effective than proteases. Tri-, and tetrapeptides, chlorogenic acids, and dihydroxy coumarins were also present in the soluble extracts of fermented DFR. The antioxidant characteristics of treated DFR were evaluated by the in vitro assays. Substitution of >5% of wheat flour with untreated DFR significantly reduced bread volume and crumb porosity; however, these adverse effects were mitigated by using fermented DFR. The highest bread volume (1845 cm^3^) and porosity (78.308%) were observed in bread produced by the substitution of 5% wheat flour with pomace that underwent enzymatic hydrolysis and ultrasound treatment. The substitution of flour with DFR significantly increased the antioxidant characteristics of bread samples and the substances generated during the in vitro digestion. It may be concluded that rowanberry pomace ingredients may improve bread nutritional quality and assist in the sustainable use of fruit processing by-products, which, together with the proper selection of pomace processing before its application in bread, may be considered as a strength of our study. Further studies should focus on a comprehensive sensory, techno-economic evaluation, and life cycle assessment of the technology to support its upscaling and commercialization.

## Figures and Tables

**Table 1:** Bread formula.

Ingredients	The Amount of Added Rowanberry Pomace, %			
	0 (Control)	5	7.5	10
Flour, g	360	342	333	324
Pomace, g	0	18	27	36
Water, mL	200	210	215	220
Yeast, g	2.87	2.87	2.87	2.87
Salt, g	3.79	3.79	3.79	3.79

**Table 2:** Proximate composition (g/100 g) and particle distribution in the pomace.

Component	Before SFE-CO_2_	After SFE-CO_2_
Moisture	3.80 ± 0.08 ^b^	3.48 ± 0.12 ^a^
Lipids	4.76 ± 0.02	nd
Proteins	5.97 ± 0.04 ^a^	6.36 ± 0.07 ^b^
Minerals	2.30 ± 0.02 ^a^	2.56 ± 0.01 ^b^
Soluble dietary fiber	6.56 ± 0.63 ^a^	8.36 ± 0.20 ^b^
Insoluble dietary fiber	46.57 ± 0.36 ^b^	43.04 ± 1.22 ^a^
Other carbohydrates *	30.04	36.20
Particle size by volume, μm	335.6 ± 17.6 ^a^	302.5 ± 21.2 ^a^
Particles by surface area, μm	101.4 ± 1.7 ^b^	84.96 ± 3.75 ^a^
Polydispersity index (span)	2.48 ± 0.14 ^a^	2.49 ± 0.07 ^a^

* calculated by subtracting other components from the total pomace mass of 100 g. Particle size by volume corresponds to D_[4,3]_, and particle size by surface area corresponds to D_[3,2]_, as determined by laser diffraction. Values are expressed as mean ± standard deviation, *n* = 3. Different lowercase superscript characters indicate significant differences between values within rows (Student’s *t*-test, *p* < 0.05). nd: not determined.

**Table 3:** The percentage yields of soluble substances and dietary fiber in the insoluble residues after treatment with ultrasound (US) and various enzyme concentrations.

Method	Soluble Substances, %		Dietary Fiber in the Dried Solids, %	
	No US	With US	No US	With US
Alkaline extraction	21.72 ± 0.70 ^a^	-		
US-assisted extraction	-	24.64 ± 0.56 ^b^		
Protease from *A. oryzae* (0.0048)	39.80 ± 0.98 ^cA^	41.34 ± 0.14 ^dB^	76.91 ± 1.50 ^dB^	68.90 ± 0.59 ^bA^
Protease from *A. oryzae* (0.0072)	39.15 ± 1.38 ^cA^	41.21 ± 0.17 ^dB^	77.10 ± 0.45 ^deB^	72.01 ± 0.58 ^cA^
Protease from *B. licheniformis* (0.01)	38.23 ± 0.81 ^cA^	40.76 ± 0.24 ^cdB^	78.75 ± 1.58 ^eB^	67.98 ± 0.20 ^aA^
Protease from *B. licheniformis* (0.02)	42.43 ± 0.34 ^eA^	43.23 ± 0.16 ^fA^	75.80 ± 1.43 ^dB^	72.30 ± 1.27 ^cA^
Viscozyme L (0.06)	47.48 ± 0.36 ^gA^	48.94 ± 0.29 ^hB^	70.01 ± 1.01 ^bcB^	67.38 ± 0.20 ^aA^

Values are expressed as mean ± standard deviation, *n* = 3. Different lowercase superscript letters denote significant differences between the columns of soluble substances and dried solids, whereas different uppercase superscript letters denote significant differences between treatments (No US, with US) in the rows of soluble substances and dried solids (*p* < 0.05).

**Table 4:** Tentative identification of compounds in soluble extracts after the hydrolysis with proteases.

Nr.	RT	*m*/*z*	Molecular Formula	Intensity *		Compound
				Ao	Bl	
1.	0.6–0.6	205.0690	C_6_H_10_N_3_O_5_	7.1 × 10^5^	7.3 × 10^5^	NI **
2.		387.1474	C_14_H_22_N_6_O_5_S	0.3 × 10^5^	-	Tetrapeptide (His Ala Gly Cys) *
3.	0.7–0.7	281.0868	C_10_H_16_O_9_	0.6 × 10^5^	0.8 × 10^5^	6-O-(3-Carboxypropanoyl)-α-D-galactopyranose
4	0.8–0.8	234.1333	C_9_H_13_N_8_	-	2.1 × 10^4^	NI **
5.	1.2–1.3	149.0810	C_6_H_12_O_4_	0.8 × 10^5^	0.9 × 10^5^	Mevalonic acid
6.	1.2–1.3	311.1333	C_12_H_22_O_9_	-	0.2 × 10^5^	Disaccharide
7.	1.2–1.3	328.1602	C_13_H_21_N_5_O_5_	0.2 × 10^5^	0.2 × 10^5^	Tripeptide (Thr-His-Ala) ***
8.	1.2–1.3	441.1965	C_19_H_28_N_4_O_8_	0.1 × 10^5^	-	Tetrapeptide (Ser-Thr-Tyr-Ala) ***
9.	1.2–1.3	621.2604	C_32_H_36_N_4_O_9_	0.29 × 10^5^	0.3 × 10^5^	Tetrapeptide (Tyr Tyr Phe Glu) *
10.	2.2–2.2	163.0390	C_9_H_6_O_3_	3.6 × 10^4^	3.8 × 10^4^	Hydroxycoumarin ****
11.	2.2–2.2	355.1022	C_16_H_18_O_9_	0.9 × 10^4^	0.8 × 10^4^	Caffeoylquinic acid *****
12.	2.5–2.5	113.0600	C_6_H_8_O_2_	2.5 × 10^5^	1.0 × 10^5^	Sorbic acid
13.	2.5–2.5	590.2729	C_30_H_35_N_7_O_6_	-	1 × 10^5^	Tetrapeptide (Trp-Trp-Gln-Ala) *
14.	2.8–2.9	163.0389	C_9_H_6_O_3_	3.4 × 10^4^	-	Hydroxycoumarin ****
15.	2.8–2.9	355.1025	C_16_H_18_O_9_	1.2 × 10^4^	2.1 × 10^4^	Caffeoylquinic acid *****
16.	2.8–2.9	487.2512	C_20_H_34_N_6_O_8_	0.3 × 10^4^	-	Tetrapeptide (ProLys-Gln-Asp) *
17.	3.0–3.1	163.0390	C_9_H_6_O_3_	2.0 × 10^4^	-	Hydroxycoumarin ****
18.	3.0–3.1	355.1022	C_16_H_18_O_9_	0.4 × 10^4^	0.7 × 10^4^	Caffeoylquinic acid *****

RT: retention time; Ao: *A. oryzae*; Bl: *B. licheniformis*; * Expressed as arbitrary units ** Not identified *** exact structure of peptide is not established; **** the site of hydroxy group is not known; ***** the site of ester link between caffeic and quinic acids is not known.

**Table 7:** Effect of defatted rowanberry pomace on the quality characteristics of bread.

Amount, %	Volume, cm^3^	Porosity, %	Moisture, %
0	1568 ± 25 ^d^	73.27 ± 1.38 ^cd^	40.27 ± 0.55 ^a^
5	1545 ± 49 ^cd^	72.55 ± 2.73 ^c^	41.69 ± 0.66 ^b^
5 *	1640 ± 60 ^e^	73.66 ± 3.79 ^cd^	42.44 ± 0.08 ^bc^
5 *#	1845 ± 64 ^f^	78.30 ± 0.58 ^d^	41.93 ± 1.51 ^b^
7.5	1453 ± 57 ^c^	67.39 ± 0.44 ^b^	42.03 ± 0.67 ^bc^
7.5 *	1498 ± 46 ^c^	71.59 ± 0.18 ^bc^	41.73 ± 0.23 ^b^
7.5 *#	1520 ± 57 ^cd^	71.90 ± 2.23 ^c^	42.28 ± 0.24 ^bc^
10	1153 ± 47 ^a^	63.33 ± 0.95 ^a^	42.46 ± 0.30 ^bc^
10 *	1213 ± 21 ^a^	63.51 ± 1.35 ^a^	42.72 ± 0.19 ^c^
10 *#	1285 ± 35 ^b^	68.57 ± 1.97 ^b^	42.03 ± 0.35 ^bc^

* The pomace was treated (hydrolyzed) with Viscozyme L; *# The pomace was treated with Viscozyme L and ultrasound. Values are expressed as mean ± standard deviation, *n* = 3. Different lowercase superscript characters indicate significant differences between values within columns (one-way ANOVA followed by Tukey’s post hoc test, *p* < 0.05).

**Table 8:** Antioxidant characteristics of rowanberry pomace and bread.

Product	TPC, mg GAE/g	ABTS, mg TE/g	ORAC, mg TE/g
Pomace			
Before SFE-CO_2_	15.07 ± 0.33 ^A^	37.16 ± 3.26 ^A^	4.93 ± 0.07 ^A^
After SFE-CO_2_	16.88 ± 0.80 ^B^	32.64 ± 3.01 ^A^	5.19 ± 0.24 ^A^
Bread			
Control	0.57 ± 0.01 ^a^	2.59 ± 0.22 ^a^	0.27 ± 0.02 ^a^
5% pomace after SFE-CO_2_	1.11 ± 0.03 ^b^	3.89 ± 0.23 ^b^	0.64 ± 0.06 ^b^
7.5% pomace after SFE-CO_2_	1.32 ± 0.01 ^c^	4.98 ± 0.19 ^c^	0.78 ± 0.04 ^c^
10% pomace after SFE-CO_2_	1.58 ± 0.04 ^d^	6.20 ± 0.34 ^d^	1.38 ± 0.17 ^d^

Values are expressed as mean ± standard deviation, *n* = 3. Different uppercase superscript letters indicate significant differences within pomace columns, while different lowercase superscript letters indicate significant differences within bread columns (one-way ANOVA followed by Tukey’s post hoc test, *p* < 0.05). Rowanberry pomace significantly increased the antioxidant potential of bread. The highest content of phenolic compounds was determined in bread in which part of the flour was replaced with 10% rowanberry pomace. Compared with the control, the TPC increased by 2.77 times, ABTS by 2.39 times, and ORAC by 5.11 times. The results show that the greater the proportion of flour replaced with rowanberry pomace, the greater the increase in antioxidant activity.

**Table 9:** Antioxidant characteristics of bread at different digestion phases.

Digestion Phase	TPC, mg GRE/mL	ABTS, mg TE/mL	ORAC, mg TE/mL
Control			
Oral (I)	0.10 ± 0.01 ^a^	0.24 ± 0.01 ^a^	0.39 ± 0.01 ^a^
Stomach (II)	0.72 ± 0.03 ^a^	1.04 ± 0.08 ^a^	3.04 ± 0.03 ^a^
Intestines (III)	0.50 ± 0.01 ^a^	2.18 ± 0.07 ^a^	2.12 ± 0.01 ^a^
With 5% fermented pomace after SFE-CO_2_ + US			
Oral (I)	0.15 ± 0.01 ^b^	0.38 ± 0.02 ^b^	0.62 ± 0.10 ^b^
Stomach (II)	0.79 ± 0.01 ^b^	2.58 ± 0.07 ^b^	3.22 ± 0.07 ^b^
Intestines (III)	0.60 ± 0.01 ^b^	2.29 ± 0.03 ^a^	2.23 ± 0.03 ^b^

Values are expressed as mean ± standard deviation, *n* = 3. Different superscript characters indicate significant differences between values separately within each assay and digestion phase (Student’s *t*-test, *p* < 0.05).

## Data Availability

The original contributions presented in this study are included in the article/Supplementary Materials. Further inquiries can be directed to the corresponding author.

## Supplementary Materials

The following supporting information can be downloaded at https://www.mdpi.com/article/10.3390/foods15091494/s1, References [24,51,59,60,61,62,63] are cited in the supplementary materials.
