# Supplementary material for: Ultrasound and Enzyme-Assisted Development of Advanced Ingredients from Rowanberry (Sorbus aucuparia L.) Pomace and Its Application in Bread
Source: Foods. 2026 Apr 24;15(9):1494. doi: 10.3390/foods15091494 (PMC13163428; doi:10.3390/foods15091494)
Supplement: Supplementary file 1 [file foods-15-01494-s001.zip › foods-4251769-supplementary.pdf]

Ultrasound and Enzyme-Assisted Development of Advanced Ingredients from Rowanberry (*Sorbus aucuparia* L.) Pomace and Its Application in Bread

Simona Ražanaitė, Laura Jūrienė, Rita Kazernavičiūtė, Michail Syrpas and Petras Rimantas Venskutonis *

Materials and Methods

1. Protein fractionation by the Osborn method (subsection 2.4.1)

Albumins were extracted from 12.5 g of defatted pomace with 250 mL of distilled water by stirring for one h in a shaker MyLab – Platform Shaker Universal PSU 20 (BioSan, Riga, Latvia). The slurry was centrifuged for 10 min at 4800 rpm in a Velocity 18R centrifuge (Dynamica, Livingston, UK). The supernatant containing albumins was poured off, and the remaining sediment was washed twice by shaking with 62.5 mL of water for 30 min and 15 min, respectively, then centrifuged under the same parameters and poured off into the 1st solution. Finally, the mixture was filtered through a Buchner funnel with a Whatman filter under a water pump, creating a vacuum.

For globulins, the precipitate remaining after albumin extraction was stirred in a shaker with 125 mL of 0.5 M NaCl for 30 min, then centrifuged for 10 min at 4800 rpm; the supernatant (globulin extract) was poured off. The precipitate was washed twice (15 min each) with 0.5 M NaCl and collected as described for albumins.

For prolamins, the precipitate remaining after globulin extraction was stirred for 30 min in a shaker with 125 mL of 70% ethanol and centrifuged for 10 min at 4800 rpm. Other steps were similar to those for globulins.

For gliadins, the precipitate remaining after prolamin extraction was stirred in a shaker for 30 min with 125 mL of NaOH solution (pH 11) at 4800 rpm speed.

All filtered supernatants were lyophilized in a MAXI DRY LYO freeze dryer (Heto LAB Equipment A/S, Allerød, Denmark), and the yield of different fractions was determined by weighing. The lyophilised extracts were stored in sealed containers in a dark, cool place.

2. Extraction of soluble substances (section 2.4.2)

2.1. Alkaline-acid extraction

Twenty-five g of defatted pomace were mixed with 250 mL of distilled water (ratio 1:10), adjusted to pH 9.5 with 2 M NaOH, incubated for two h in a water bath WNB 22 (Memmert, Germany) at 45 °C under shaking, and centrifuged for 30 min at 4800 rpm. The separated solid phase was dried at 60 °C in an Ecocell drying oven (MMM Medcenter, Planegg, Germany), and the liquid phase was acidified to pH 4 with HCl, kept at 4 °C for 24 hours, and centrifuged under the same conditions. The resulting solution was lyophilized, while the precipitates were dried at 60 °C to determine the yields.

2.2. Ultrasound-assisted extraction (UAE)

Defatted pomace was mixed with distilled water at a ratio of 1:10, then exposed to ultrasound using an ultrasonic homogenizer UP200Ht (Hielscher, Teltow, Germany) for 65 min at 200 W and 30–55 °C, and

centrifuged for 30 min at 4800 rpm. The separated solid fraction was dried at 60 °C, the liquid supernatant was lyophilized, and the yields were determined.

2.3. Enzyme-assisted extraction (EAE)

The complex of cellulases, Viscozyme L, containing cellulase, xylanase, and hemicellulase from *Aspergillus* sp. was used for the hydrolysis of cell wall polysaccharides and consequent release of bound proteins from their matrix. Proteolytic enzymes from *Bacillus licheniformis* and *Aspergillus oryzae* were used to proteolyze proteins into shorter polypeptides or amino acids.

Defatted pomace was mixed with distilled water at a 1:10 ratio, and the pH was adjusted to 4.5 for Viscozyme L and to 9 for proteolytic enzymes with 2 M NaOH. Then, the recommended amount of enzyme [24] was added (in mL/g pomace): Viscozyme L – 0.06; proteases from *B. licheniformis* – 0.01 or 0.02; proteases from *A. oryzae* – 0.0048 or 0.0072. The prepared samples were incubated in a vibrating water bath (200 rpm) at 50 °C for 117 min (Viscozyme L) or 68 min (proteolytic enzymes). After increasing the pH to 9.5, the samples were kept at 45 °C for 30 min. After protein hydrolysis, the samples were filtered through filter paper and centrifuged for 30 min at 4800 rpm. The separated solid phase was dried at 60 °C, while the liquid supernatant was lyophilized to determine the yields. All dried fractions were stored in tightly closed bags in a dark, dry place until further analysis.

2.4. Enzyme-ultrasound assisted extraction (EAE-UAE)

The treatment of pomace samples with enzymes and ultrasound was performed in two ways: (1) the pomace was prepared for enzymatic treatment as in 2.3 and afterwards subjected to ultrasound as in 2.2.; (2) the pomace was treated with ultrasound (65 min, 200 W, 50 °C) and afterwards the pH was adjusted to 4.5 with 2 M NaOH, Viscozyme L (0.06 mL/g) added and incubated for three h at 45 °C in a vibrating water bath. After enzyme-ultrasound or ultrasound-enzyme treatments, proteolytic hydrolysis was performed as in 2.3.

3. Determination of soluble and insoluble fiber content (sections 2.3 and 2.4.2)

The content of soluble and insoluble fiber was determined using Fibertec 1023 (FOSS, Denmark) according to the reference method AOAC 991.43. Rowanberry pomace (0.25 g) was weighed into 400 mL plastic containers, and 10 mL of MES-TRIS (pH 8.2) buffer solution was added and mixed. 12.50 μL of heat-stable α-amylase solution was added to the samples. The plastic containers were covered with foil and placed in a WNB 22 water bath (Memmert, Germany), where they were incubated and shaken for 30 min at 98–100 °C. After the specified time, the samples were removed and cooled to room temperature, and the water bath was cooled to 60 °C. The walls of the plastic container were cleaned with a spatula, then rinsed with 5 mL of distilled water.

Once the required temperature was reached in the water bath, 25 μL of protease solution was added to plastic containers, which were covered with foil and placed in a water bath, where they were kept and shaken for 30 min at 60 °C. After the specified time, the samples were removed and cooled to room temperature. The pH was then adjusted to 4.1–4.8 with 5% NaOH, and 50 μL of amyloglucosidase solution was added. The containers were covered with foil, placed in a water bath, and kept there while they were shaken for 30 min at 60 °C.

Glass crucibles are used to determine the amount of fiber. Before use, they were heated to constant mass. One gram of celite was weighed into the crucibles. Distilled water was added, the crucibles were mixed, and, under vacuum, crucibles with a celite layer were prepared. The crucibles were placed in a drying oven at 105 °C until constant weight.

4. Determination of bread volume, crumb porosity, and moisture (subsection 2.7.3)

In general, the guidelines provided in Baking Quality Methods, which are available on-line (https://www.cerealsgrains.org/resources/Methods/Pages/10BakingQuality.aspx) were used with slight modifications. An overfilled millet groats container was used to determine the volume of a baked good. The millet in the container was leveled with a ruler so that it was precisely equal to the container's volume. The excess was removed, and the groats were transferred to another container. The bread loaf was placed in an empty container, the millet was poured in, and the ruler was used to smooth the surface. The millet that no longer fills the container corresponds to the volume of the baked good being measured. The tested bread loaf displaces its volume of millet, which is measured with a measuring cylinder. The volume of the baked good is expressed in cm^3.

To measure the porosity, the cooled bread was cut in half, and cylindrical pieces of crumb were cut out, no closer than 1 cm to the crust. It was pushed out of the Zhuravliov cylinder with a wooden plunger to the device's support and cut off at the mark with a sharp knife. The 27 cm^3 volume pieces were weighed on an analytical balance with an accuracy of 0.01 g, and bread porosity calculations were performed according to the formula: $A = (V-G/d)/V \times 100\%$, where V – total volume of cutouts, cm^3; G – total mass of cutouts, g; d – relative density of non-porous pulp (for bread made of wheat flour 550D – 1.31).

The moisture content was determined by the Moisture Analyser MB64 60G (VWR, Milan, Italy). Briefly, ~1 g of bread crumb was weighed into the foil plate, the device settings were selected according to the manufacturer's instructions, and dried at 105 °C to a constant mass. Three replicate determinations were performed for all characteristics.

5. *In vitro* gastrointestinal digestion of bread by global antioxidant response (GAR) method (section 2.7.4.)

The method of Miller et al. (1981) was modified by including an oral step. Three digestive phases (mouth, stomach, and intestines) were analyzed, with three replicates for each phase. For this purpose, 9 test tubes with 1 g of dried and ground (0.2 mm) bread powder were mixed with 7 mL of α-amylase solution (32.5 mg in 25 mL of $CaCl_2$) and shaken for 30 min at 200 rpm in an Orbital Shaker – Incubator ES-20/60 (BioSan, Riga, Latvia) at 37 °C. Afterwards, three tubes were removed, placed in boiling water for 4 min, transferred to ice-cold water, centrifuged for 30 min at 4800 rpm, and filtered through a 0.2 μm pore size filter.

For the stomach phase, the remaining six tubes were acidified to pH 2 with 1 M HCl, then mixed with 1.2 mL of pepsin solution (0.4 g in 2.5 mL of 0.1 M HCl), and stirred for two hours at 37 °C. Afterwards, three tubes were removed, heated in boiling water for 4 min to inactivate the enzyme, transferred to ice-cold water, centrifuged, and filtered.

For the intestinal phase, three tubes after digestion in the stomach were alkalized to pH 6 with 1 M $NaHCO_3$, mixed with 3 mL of pancreatin bile extract (0.1 g of pancreatin and 0.625 g of bile extract dissolved in 25 mL of 0.1 M $NaHCO_3$), the pH adjusted to 7.5, incubated for two hours at 37 °C, placed in boiling water for 4 min, cooled in iced water, centrifuged and filtered. The obtained extracts were further analyzed by the in vitro antioxidant assays.

6. Determination of antioxidant properties in extracts and solid fractions (section 2.8.)

6.1. $ABTS^{\bullet+}$ scavenging (decolorization) assay

The method of Re et al. (1999) was used with minor modifications. Briefly, a phosphate-buffered saline (PBS, pH 7.4) solution was prepared by dissolving 8.18 g NaCl, 0.27 g KH_2PO_4, 1.42 g Na_2HPO_4, and 0.15 g KCl in 1 L of distilled water. The initial $ABTS^{\bullet+}$ solution was prepared by dissolving 0.05487 g ABTS in a 50 mL volumetric flask with PBS, adding a solution of K2S2O8 (0.0038 g/200 μL of distilled water), and incubating for 15–16 h in the dark. A working solution is prepared by diluting the initial

solution with PBS and measuring absorbance on a Spectronic Genesys 8 (Thermo Scientific, USA) to obtain an optical density of 0.800 ± 0.010 at 734 nm.

For extracts, 25 μL of the extract or control sample (distilled water) was mixed with 1500 μL of the working solution, vortexed for 15 s, incubated in the dark for 2 hours, and the absorbance was read at 734 nm. Reference Trolox solutions of various concentrations were used to produce a calibration curve: y = 0.0657x + 1.4911; R2 = 0.999.

For solids, 10 mg of the fraction or microcrystalline cellulose (control) were mixed with 25 μL of methanol, and 1500 μL of the working solution, vortexed for 15 s, then left for two hours, placed in a shaker (250 rpm), centrifuged (8500 rpm, 10 min) and the absorbance of the clear liquid measured at 734 nm. The calibration curve was constructed: y = 0.0625x – 2.804; R2 = 0.997. Antioxidant capacity is expressed as Trolox equivalents (mg TE/g DM).

6.2. Determination of total phenolic compounds (TPC)

The method described by Singleton et al. (1999) was used. The Folin-Ciocalteu solution was prepared by diluting the reagent (Sigma-Aldrich, Switzerland) with distilled water in a ratio of 1:9. For extracts, 150 μL of the sample or distilled water (control) were mixed with 750 μL of Folin-Ciocalteu working solution, after 3 min, 600 μL of 7.5% Na_2CO_3 admixed, left for two hours in the dark, and the absorbance measured at 760 nm. A solution of gallic acid was used to construct a calibration curve: y = 0.0117x + 0.0025; R2 = 0.9998.

For the solids, 10 mg of the sample or microcrystalline cellulose (control) were mixed with 150 μL of distilled water and 750 μL of Folin-Ciocalteu working solution; after 3 min, 600 μL of 7.5% Na_2CO_3 solution added, thoroughly mixed for 15 s, transferred to a shaker for two hours in a dark, and centrifuged for 10 min at 8500 rpm. The absorbance of the transparent liquid was measured at 760 nm. The calibration curve was constructed: y = 0.0121x + 0.0298; R2 = 0.9934. The TPC was expressed as gallic acid equivalents (mg GAE/g).

6.3. Determination of oxygen radical absorbance capacity (ORAC)

The method of Prior et al. (2003) was used with minor modifications. Fluorescein solutions (14 μmol/L) were prepared by dissolving in PBS: 0.0225 g in 50 mL (S1); 125 μL of S1 in 25 mL; 800 μL of S2 in 50 mL (working solution). For the extracts, 25 μL of the extract or distilled water (control) sample were poured into a black opaque 96-well microplate, 150 μL of the S3 added to the wells with an automatic multichannel pipette, incubated for 15 min at 37 °C, 25 μL of 240 mmol/L azobis (AAPH) solution (0.125 g in 1.92 mL of PBS) added to each well; the microplate placed in a FLUOstar Omega reader spectrophotometer (BMG Labtech, Ortenberg, Germany) and 120 cycles of measurements were performed at 37 °C with excitation at 485 nm and emission at 520 nm. Trolox solutions of various concentrations are used to construct a calibration curve: y = 0.1394x - 0.7395; R^2 = 0.9987.

For the solids, 10 mg of the fraction or microcrystalline cellulose (control) were mixed in an Eppendorf with 150 μL of PBS solution and 900 μL of S3; vortexed 15 s, shaken for 60 min in a dark, centrifuged (8500 rpm, seven min), 175 μL of the clear supernatant transferred with an automatic micropipette to a 96-well plate, incubated for 15 min at 37 °C, 25 μL of AAPH solution added to each well and 180 cycles of measurements performed. The calibration curve was constructed: y = 0.1325x + 3.5534; R^2 = 0.9966. The area under the calibration curve was calculated by integrating the relative fluorescein curve, and the ORAC was expressed in mg TE/g.

6.4. Preliminary Evaluation of Sensory Properties

A complete scope scientific evaluation of the effects of rowanberry pomace ingredients on the sensory quality of bread was not performed in this study. The students and junior staff from the Department were asked to verbally describe bread samples, indicating the differences between them

without assigning points. The evaluators noted that when the untreated pomace content increases to 10%, the bread acquires stronger notes of bitterness, sourness, and aroma and receives a lower overall evaluation. The use of ultrasound- or enzyme-treated pomace mitigated these adverse effects, and bread produced by substituting wheat flour with 5–7.5% pomace received a descriptive overall assessment similar to that of the control bread. Based on these preliminary evaluations, it may be concluded that the overall sensory evaluation of bread produced with up to 7.5% pomace does not significantly differ from the control bread. Moreover, the sensory properties of bread containing 5% ultrasound/Viscozyme-treated pomace were better than those of the other samples. At this concentration, the rowanberry pomace slightly increased the bread's sourness and imparted some fruit aroma. Evident colour changes may be observed (Figure 3). Colour is a crucial factor in consumers' decision-making during shopping. It may be evaluated using sensory and/or instrumental methods, e.g., by measuring Lab colour characteristics and/or by calculating browning and whiteness indices for bread crust and crumb, respectively. In our case, these indices would be less critical to practical consumer acceptance because the effect of pomace additives on colour is evident and can be clearly observed by the naked eye (Figure 3). Instrumental colour measurements would be essential when applied ingredients are less colourful and do not visibly affect the final bread, e.g., as previously reported in using alfalfa seeds [59].

The positive effects of rowanberry pomace on other bread properties may also be expected. For instance, Samilyk et al. [60] studied the impact of S. aucuparia powder on the shelf life of wheat bread and reported that adding 20% rowanberry powder at least doubled the shelf life. However, a high rowanberry powder content harms bread porosity and sensory properties. At the same time, the addition of 10% powder did not impart any defects in the taste, odor, or shape of bread. These findings support the possibility of replacing wheat flour with rowanberry pomace ingredients without any inferior effects on bread quality.

It may be noted that the impact of berry powder on the sensory quality of bread depends both on the berry product and treatment. For instance, an acceptable level of haskap berry (Lonicera caerulea L.) pomace was only 2% [51], whereas goji berry substitution up to 20% was preferred by the consumers [61]. Statistical models for sensory preference evaluation and correlation with wheat bread crumb structure and color indicated that the best acceptance of the fibre-rich elderberry by-product was 7.0% [61]. In addition, it may be noted that due to consumer awareness of health and sustainability issues, consent to use pomace in food products was remarkably high (>90%) as shown by the study performed in Germany, Italy, Spain, Sweden, and the UK, using savoury crackers produced with 20% of blackcurrant pomace [62]. For instance, blackcurrant pomace inhibited starch-digestive enzymes, which may reduce glucose release, and thereby counteract the hyperglycaemic effect after consumption of bread and other bakery products [63].
